# Cytotoxic and Apoptogenic Activity of Bryonia aspera Extract on Pre-B Acute Lymphoblastic Leukemia Cell Lines

**Published:** 2018-07-01

**Authors:** Sorur Yazdanpanah, Somayeh Esmaeili, Davood Bashash, Nasrin Dehghan Nayeri, Mohammad Esfini Farahani, Ahmad Gharehbaghian

**Affiliations:** 1 Department of Laboratory Hematology and Blood Bank, School of Allied Medical Sciences, Shahid Beheshti University of Medical Sciences, Tehran, Iran; 2 Traditional Medicine and Materia Medica Research Center (TMRC), Shahid Beheshti University of Medical Sciences, Tehran, Iran

**Keywords:** Acute lymphoblastic leukemia, *Bryonia aspera*, Prednisolone, Apoptosis, Combination therapy

## Abstract

**Background:** The natural products and conventional chemotherapeutic drug combinations are believed to increase cure rates of anticancer treatment while reducing its toxicity. The current study investigates cytotoxic and apoptogenic effects of methanolic extract of *Beryonia aspera*, and also synergistic effects of this extract and Prednisolone on acute lymphoblastic leukemia cell lines.

**Materials and Methods: **The under study populations were NALM-6 and REH cell lines. Cells were treated by Prednisolone and *B. aspera *extract alone and in combination. The effect of the drugs on survival and apoptosis were examined using MTT and flow cytometry, respectively. Moreover, the effects of the drugs on the mRNA expression levels of *Bax* and *Bcl-2* were studied using RQ-PCR. Finally, both the transcriptional and enzymatic activity of caspase-3 were investigated by caspase-3 assay kit.

**Results: **The *B. aspera* extract induced cell growth inhibition and triggered apoptosis in a dose- and time-dependent manner. Real-time PCR analysis of apoptotic target genes revealed that this agent shifted the ratio of the death promoter to death repressor genes via alteration of *Bax* and *Bcl-2* expression levels. These changes resulted in caspase-3 activation, which led to DNA fragmentation and subsequent apoptosis. Our study has also demonstrated that the combined treatment of *B. aspera* extract with Prednisolone did not induce greater cytotoxic effect as compared to treatment series using either Prednisolone alone.

**Conclusion: **Our study demonstrated that the *B. aspera* extract has anti-leukemic properties on BCP-ALL cell lines and could be regarded as a promising agent for the treatment of ALL.

## Introduction

 B-cell precursor acute lymphoblastic leukemia (BCP-ALL) is the most common type of malignancy in children ^1^^,^^2^ . Glucocorticoids have been widely used as effective therapeutic agents for these malignancies because glucocorticoids induce growth arrest and apoptosis in B lymphocytes^   3 ^. Prednisolone, a synthetic glucocorticoid, is one of the most important pharmaceutical agents in the treatment of ALL, especially in childhood ALL^   4 ^. These agents have many side effects such as immunodeficiency, hyperglycemia, increased skin fragility, easy bruising, steroid-induced osteoporosis, etc^   5 ^. Dramatic advancement in an effective treatment for childhood ALL has led to an overall cure rate of more than 80%, providing opportunities for innovative combined-modality strategies that would increase the efficacy of treatment while reducing the toxic side-effects of current intensive regimens^   1 ^. Since the medicinal herbs due to the natural origin are more compatible than chemical drugs with living organisms and cause fewer side effects, thus, as a potential resource of new supplements for chemotherapy drugs, are paid special attention^   6 ^. According to the World Health Organization (WHO), approximately 60% of anti-tumor drugs are derived directly or indirectly from medicinal plants    7 . The selection of plants that may contain new biological agents is done, using different strategies. In the ethnomedical strategy, belief is given to oral or written information on medicinal use of the plant, and accordingly the plant is collected and evaluated  ^8^^,^^9^ . *Bryonia aspera Steven ex Ledeb.* is a species of Bryonia which belongs to the family of Cucurbitaceae, and is locally known as “Andaz”. Biological values of cucurbit plants were early recognized by traditional medicine. They were used actively as traditional herbs for treating many types of diseases and indicated to have anti-inflammatory, anti-tumor, hepatoprotective and immunomodulatory properties. Ethnopharmacological information indicates that the *B. aspera *is used in the Turkmen Sahra region, at the north of Iran, to treat cancer, gastric, hepatic and heart disorders and rheumatism^9^^,^^10^. Additionally, this plant has been reported to possess cytotoxic effects on breast cancer^   9 ^, cervix adenocarcinoma and head and neck squamous cell carcinoma cell lines^   11 ^.

The present study was conducted to investigate the therapeutic effect of methanolic extract of aerial parts of* B. aspera*, and also synergistic cytotoxic effects of this extract and Prednisolone on glucocorticoid non-sensitive (REH)^   12 ^, and partially sensitive (NALM-6)^   13 ^ acute lymphoblastic leukemia cell lines.

## MATERIALS AND METHODS

**Plant material**

The aerial parts of *B. aspera *were collected from the entrance of Pelor, Tehran province, Iran, and were identified by Mr. Moazeni, Traditional Medicine & Materia Medica Research Center, Shahid Beheshti University of Medical Sciences, Tehran, Iran. Voucher specimen of the plant (TMRC 3637) has been deposited in the herbarium of the Traditional Medicine & Materia Medica Research Center. 

**Preparation of extract**

The aerial parts of plant were air dried at room temperature, powdered and then extracted with methanol by maceration and with constant shaking for 24 h. The plant extract was then filtered and the solvent was evaporated under vacuum by means of a rotary evaporator and stored at 4°C before evaluating biological activities. 

**Cell culture and** ***B. aspera*** **extract /Prednisolone treatment**

NALM-6, REH (Human B-cell precursor acute lymphoblastic leukemia cell lines) and MDBK cells (Bovine kidney cell line) were purchased from Pasteur Institute of Iran and were cultured in RPMI 1640 medium supplemented with 2 mM L-glutamine, 10 % FBS, 100 units/ml penicillin, and 100 μg/ml streptomycin in a humidified 5 % CO_2_ incubator at 37 °C under standard cell culture conditions. Prednisolone powder was obtained from Sigma Aldrich Company (Germany). For *B. aspera* extract treatment, cells were treated with relevant amounts of *B. aspera* extract stock solution to attain concentration of 100, 150, 200, 250, 300, 350, 400 and 450 µg/ml. For Prednisolone treatment, cells were treated with relevant amounts of the Prednisolone to attain concentration of 500 nM, 1, 10, 50, and 100 M.

**MTT assay**

The MTT assay was used to evaluate the cytotoxicity^   14 ^^.^ For suspension cells (NALM-6 and REH), cells were briefly cultured at 5000/well in 96-well plate and incubated with desired concentrations of* B. aspera* extract, Prednisolone and the combination of *B. aspera* extract and Prednisolone for 24 and 48 h. After removing the media, cells were further incubated with MTT solution (5 mg/ml in PBS) at 37ºC for 3 h and the untreated cells were defined as the control group. The resulting formazan was solubilized with Dimethyl sulfoxide** (**DMSO) and the absorption was measured at 570 nm (620 nm as a reference) in ELISA reader and IC_50_ was calculated as the concentration of fractions and compounds causing a 50% inhibition of cell viability.

For adherent cells (MDBK), 10000 cells were cultured into a 96-well plate and 24 h later cells were washed and maintained with different concentrations of *B. aspera* extract for 24 and 48 h, at 37°C under 5% CO_2_ atmosphere. After removing the media, cells were further incubated with MTT solution (5 mg/ml in PBS) at 37ºC for 3 h and the untreated cells were defined as the control group. The resulting Formazan was solubilized with DMSO and the absorption was measured at 570 nm (620 nm as a reference) in ELISA reader.

**Phosphatidylserine externalization (Annexin-V assay)**

The apoptosis induced by *B. aspera* extract were analyzed by Annexin-V/PI double staining kit (eBioscience) based on the manufacturer’s instructions. Briefly, leukemic cells were cultured into 12-well cell culture plates and treated with different concentrations of *B. aspera* for 48 h and were then collected. Afterwards, 200 l of a binding buffer and then 5 l of Annexin-V were added to the cell suspension and were incubated for 10 minutes. The cells were rinsed and 200 l of the binding buffer was added. PI was added before reading the values with flow cytometry. Annexin-V positive and PI-negative cells were considered to be in early apoptotic phase and cells with Annexin-V and PI positive were considered to undergo late apoptosis or necrosis.

**RNA purification, reverse transcription, and real-time PCR amplification**

Total RNA was isolated 48 h after treatment with *B. aspera* extract using RNX plus (SinaClon, Iran). The reverse transcription (RT) reaction was performed using the revertAid First Strand cDNA Synthesis Kit (Takara BIO). The cDNA prepared was subjected to qRT-PCR on a light cycler instrument (Roche). Thermal cycling conditions included activation step for 30 s at 95°C followed by 45 cycles, including a denaturation step for 5 s at 95°C and a combined annealing/extension step for 20 s at 60°C. A melting curve analysis was applied to verify the specificity of the products, and the values for the relative quantification were calculated based on 2^-ΔΔCt^ relative expression formula. Nucleotide sequences of the primers used for qRT-PCR are shown in Table 1. 

**Table:** 

**Gene**	**Forward primer (5’-3’)**	**Reverse primer (3’-5’)**	**Size ** **(bp)**
HPRT	TGGACAGGACTGAACGTCTTG	CCAGCAGGTCAGCAAAGAATTTA	111
Bax	CGAGAGGTCTTTTTCCGAGTG	GTGGGCGTCCCAAAGTAGG	242
Bcl-2	CGGTGGGGTCATGTGTGTG	CGGTTCAGGTACTCAGTCATCC	90

*HPRT* (hypoxanthine phosphoribosyltransferase), *Bax* (Bcl-2 associated protein), *Bcl-2* (B-cell lymphoma)

**Caspase-3 enzymatic activity**

To measure the potential of *B. aspera* extract in triggering of apoptosis through up-regulation of caspase-3, cells were subjected to apoptosis analysis using caspase-3 assay kit (Sigma). This assay is based on spectrophotometric detection of the color reporter molecule p-nitroaniline (pNA) that is linked to the end of caspase-specific substrate. After treatment, cells were incubated for 48 h. Following centrifugation at 600×g for 5 min, the cell pellets were lysed and the lysates were centrifuged at 20,000×g for 10 min. In a total volume of 100 µl, 5µg of the supernatant was incubated with 85 µl of assay buffer plus 10 µl of caspase-3 substrate (Ac-DEVD-pNA) in a 96-well plate at 37ºC for 2 h. The cleavage of the peptide by caspase releases the chromophore pNA, which was quantified spectrophotometrically at a wavelength of 405 nm. The results were expressed as fold increase in caspase activity of apoptotic cells in the treated cells over that of untreated cells.

**Statistical analysis**

Experimental data are expressed by mean ± standard deviation of three independent assays. All tests were done in duplicate or triplicate. Statistical significance was calculated using paired two-tailed Student’s t-tests. Statistically different values were defined significant at *P <0.05, ** P < 0.01, and ***P <0.001.

## Results

**Reduction of cellular metabolic activity in BCP-ALL cell lines b** ***y B. aspera*** **extract**

**Figure 1:**
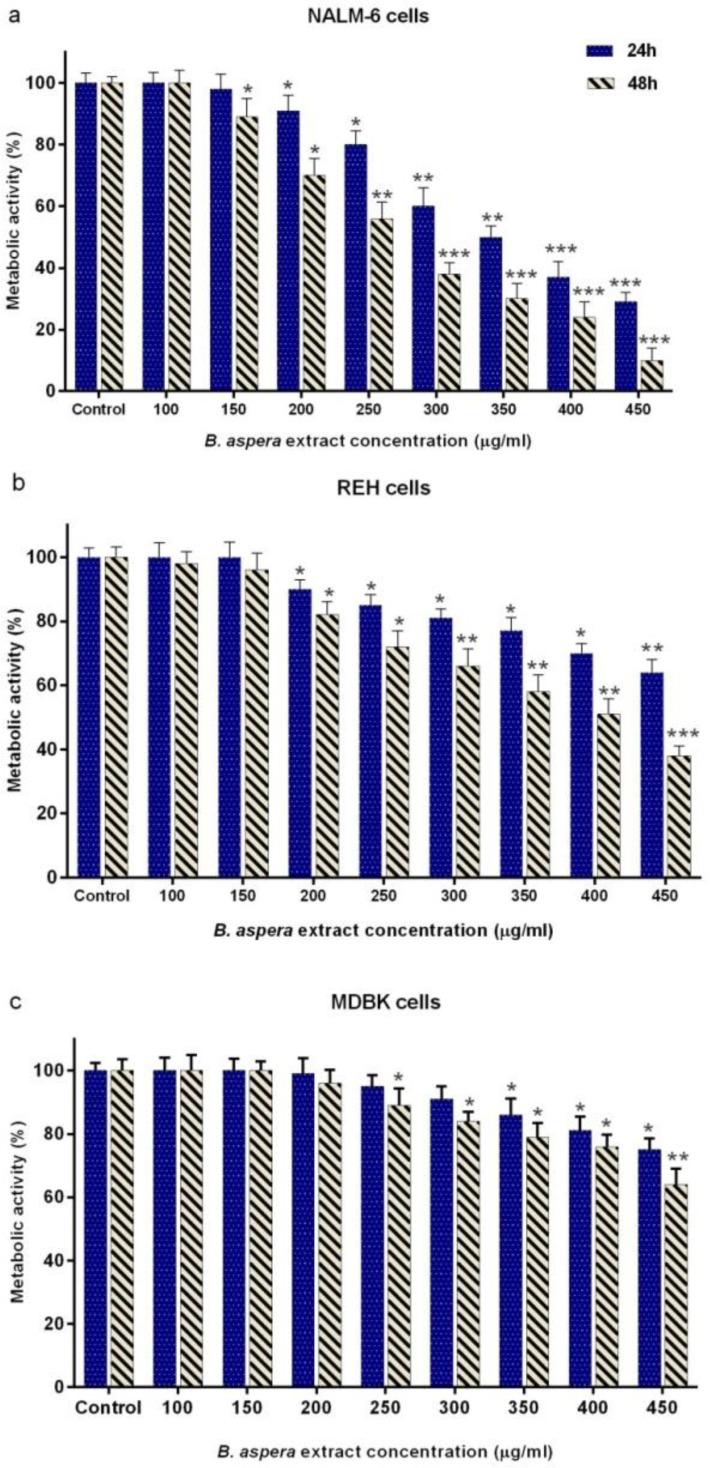
Cytotoxic effect of B. aspera extract on NALM-6, REH and MDBK cell lines. The cells were treated with different concentration of B. aspera extract and their metabolic activities were measured after 24 and 48 h incubation, using the MTT assay. Values are given as mean ±SD of three independent experiments. *P < 0.05, **P < 0.01, and ***P < 0.001 represent considerable alterations from untreated control.

The cytotoxic effects of different concentrations of *B. aspera* extract (100-450 μg/ml) on metabolic activity of NALM-6 and REH cells after 24 and 48 h treatment were evaluated with the MTT assay. As indicated in Figure 1 a and b, the extract of *B. aspera* reduced the survival of NALM-6 and REH cells in a dose- and time-dependent manner. In this study, CompuSyn software was used to calculate IC_50_. The IC_50_ values of *B. aspera* extract in NALM-6 and REH cell lines after 48 h incubation were approximately 282 and 380 μg/ml, respectively. In addition, the MDBK cells were used as the normal cell line to examine the effect of this extract on normal cells. The IC_50_ value of the *B. aspera* extract on MDBK cells was approximately 467 μg/ml after 48 h incubation (figure 1c), suggesting the lower cytotoxic effect of this extract on MDBK cells than on acute lymphoblastic leukemia cell lines.

**Induction of apoptosis in BCP-ALL cell lines by** ***B. aspera*** **extract**

**Figure 2:**
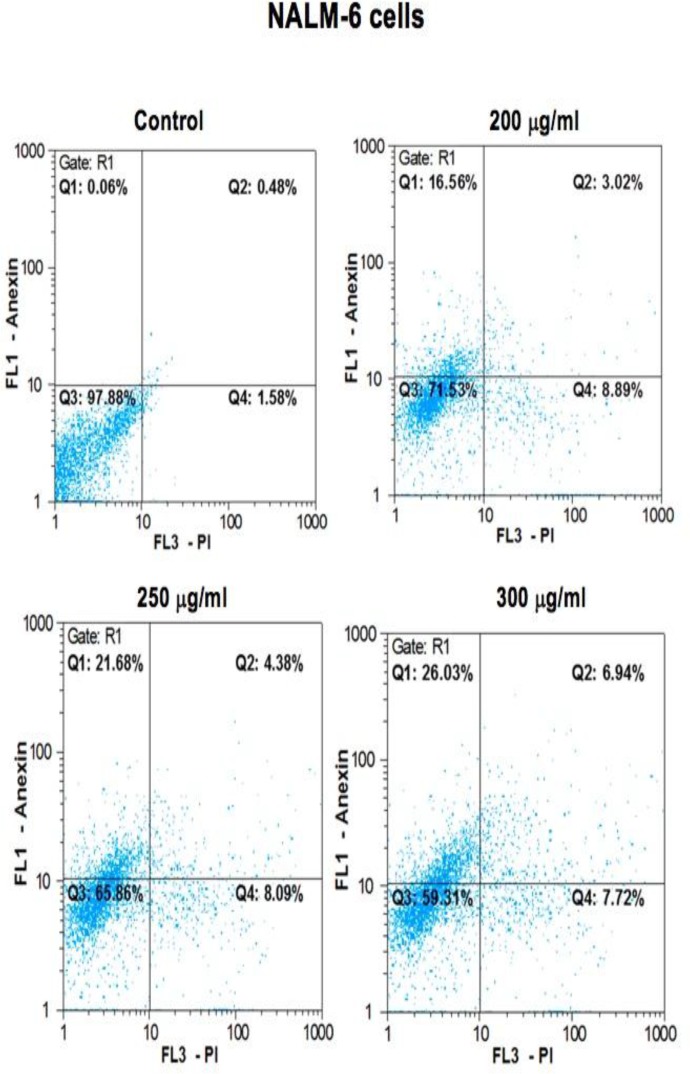
Results from the induction of apoptosis by the B. aspera extract on NALM-6 cell line. The NALM-6 cells were exposed to intended concentrations of the B. aspera extract and then the induction of apoptosis after 48 h incubation was evaluated. Flow cytometry images, Q1, Q2, Q3 and Q4 represent early apoptosis, late apoptosis, live cells, and necrotic cells, respectively.

**Figure 3:**
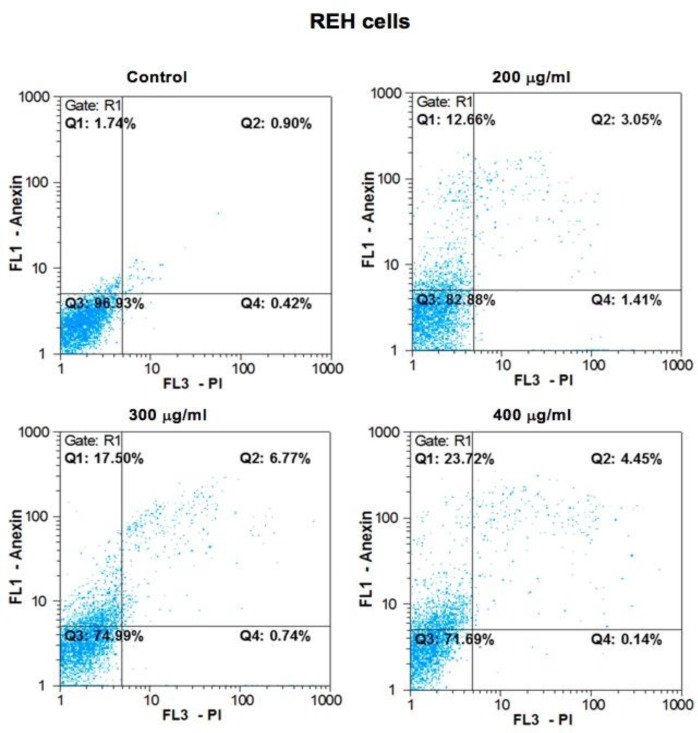
Results from the induction of apoptosis by the B. aspera extract on REH cell line. The REH cells were exposed to intended concentrations of the B. aspera extract and then the induction of apoptosis after 48 h incubation was evaluated. Flow cytometry images, Q1, Q2, Q3 and Q4 represent early apoptosis, late apoptosis, live cells, and necrotic cells, respectively.

To validate whether cell survival reduction was due to apoptosis induction, NALM-6 and REH cells were treated with intended concentrations of *B. aspera* extract, and the apoptosis index was examined through Annexin-V/PI assay after 48 h incubation, using the flow cytometry technique. Results showed that the *B. aspera* extract was capable of inducing apoptosis in BCP-ALL cell lines in dose-dependent manner. As shown in Figure 2, 200, 250 and 300 μg/ml doses of *B. aspera* extract caused 16.5, 21.6 and 26% early apoptosis on NALM-6 cells, and similar apoptogenic effects were also apparent on REH cell line as indicated in Figure 3, such that 200, 300 and 400 μg/ml doses of *B. aspera* extract caused 12.6, 17.5 and 23.7% early apoptosis on these cells.

**Increased transcription of** ***Bax*** **and reduced transcription of** ***Bcl-2*** **in BCP-ALL cell lines by** ***B. aspera*** **extract**

**Figure 4:**
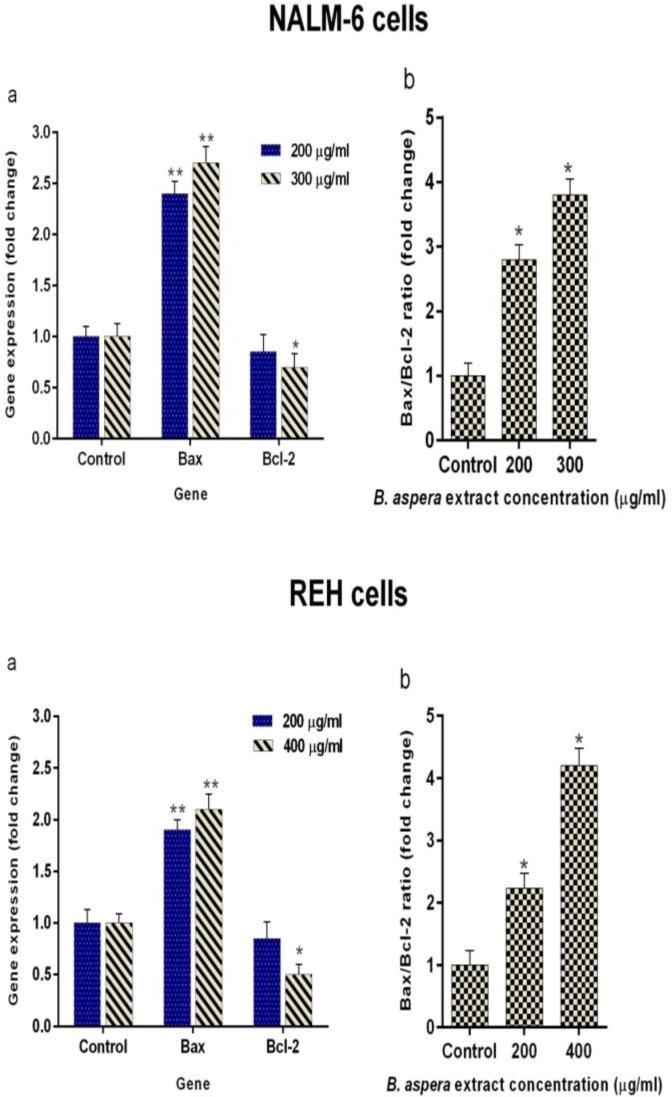
Expression of Bax and Bcl-2 genes in NALM-6 and REH cells treated with desirable concentration of B. aspera extract. (a) The NALM-6 and REH cells were exposed to the desirable concentration of the B. aspera extract, and the expression of Bax and Bcl-2 genes was examined after 48 h incubation using real-time RT-PCR. (b) Bax/Bcl-2 ration. Values are given as mean ±SD of three independent experiments. *P < 0.05 and **P < 0.01 represent considerable alterations from untreated control.

The *Bax* (pro-apoptotic) and *Bcl-2* (anti-apoptotic) proteins are the most important members of *Bcl-2* family and have a central role in regulating the programmed cell death (apoptosis)^   15 ^. Accordingly, the expression of *Bax* and *Bcl-2* genes following the treatment of cells with intended concentrations of the extract and after 48 h incubation was quantified to investigate the effect of *B. aspera* extract on the induction of cell death on NALM-6 and REH cells. The regulation of gene expression was expressed as fold differences between control and treatment groups as shown in Figure 4. Results indicated an increase in the transcription of *Bax* and a reduction in the transcription of *Bcl-2* as compared to the control. Reduce *Bcl-2* mRNA level coupled with up-regulated transcription of *Bax* was associated with an increase in *Bax*/*Bcl-2* transcriptional ratio as a result of treating NALM-6 and REH cells with *B. aspera* extract. This increase and decrease disturbed the balance between pro- and anti-apoptotic proteins in favor of pro-apoptosis factors in BCP-ALL cell lines.

**Increased transcription and enzymatic activity of caspase-3 in BCP-ALL cell lines by** ***B. aspera*** **extract**

**Figure 5:**
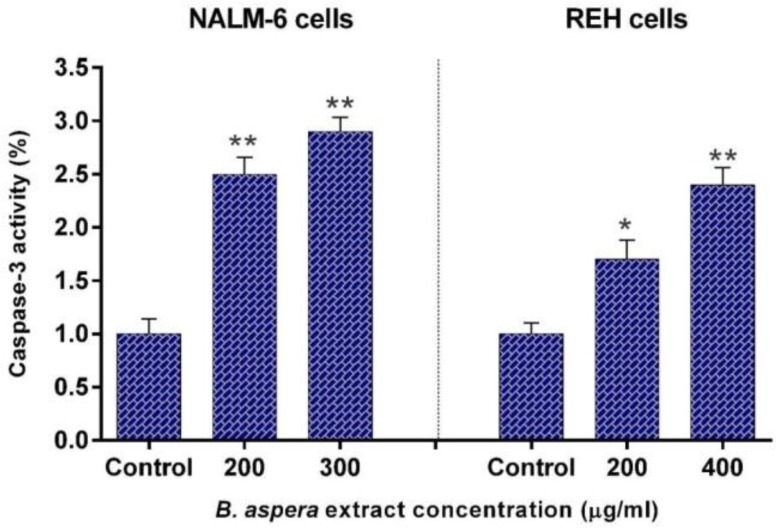
Caspase-3 activity in NALM-6 and REH cells treated by B. aspera extract. The NALM-6 and REH cells were exposed to the desirable concentration of the B. aspera extract, and the activity of caspase-3 enzyme was examined after 48 h incubation. Values are given as mean ±SD of three independent experiments. *P < 0.05 and **P < 0.01 represent considerable alterations from untreated control.

To confirm the apoptotic effects of *B. aspera* extract, the enzymatic activity of caspase-3 was also quantified. To this end, the cells were treated with desirable concentrations of the extract for 48 h, and the enzymatic activity changes of caspase-3 was evaluated, using caspase-3 assay kit (Colometric). Results indicated that the *B. aspera* extract increased the enzymatic activity of caspase-3 in BCP-ALL cells treated with this extract (Figure 5).

**The lack of synergistic effect in using** ***B. aspera*** **extract in combination with Prednisolone in BCP-ALL cell lines**

**Figure 6:**
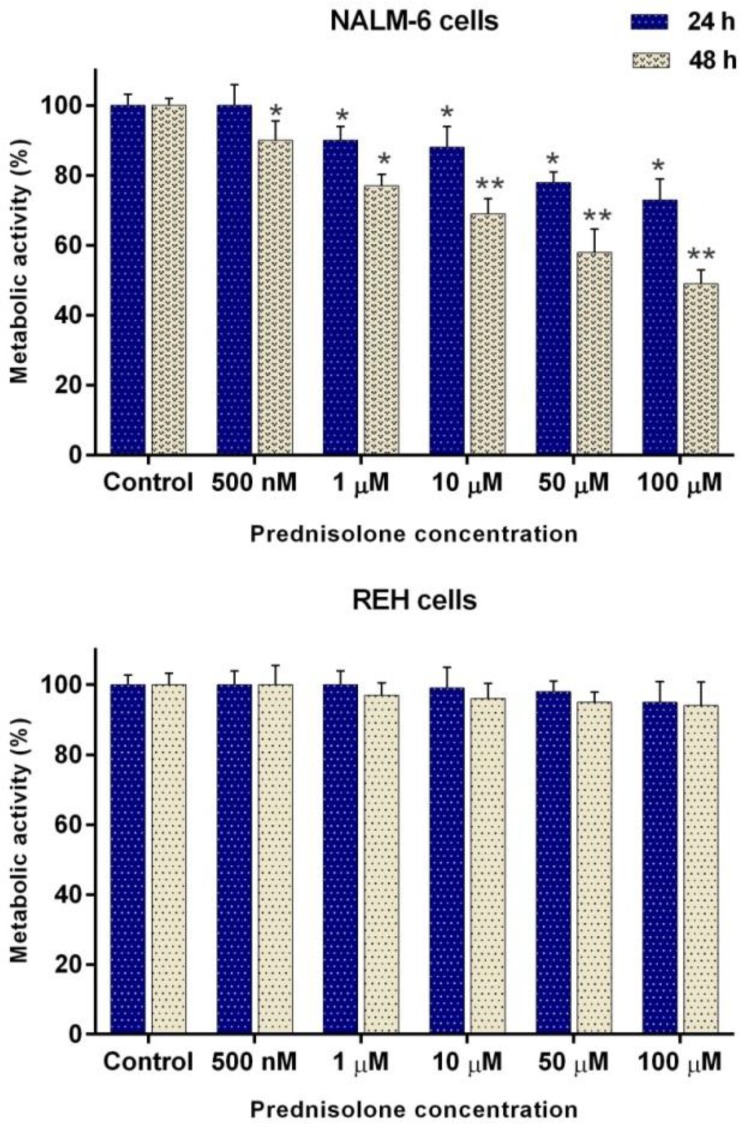
Cytotoxic effect of Prednisolone on NALM-6 and REH cell lines. The cells were treated with different concentration of Prednisolone and their metabolic activities were measured after 24 and 48 h incubation, using the MTT assay. Values are given as mean ±SD of three independent experiments. *P < 0.05 and **P < 0.01 represent considerable alterations from untreated control.

To see whether the *B. aspera* extract can increase the sensitivity of BCP-ALL cell lines to Prednisolone as a glucocorticoid drug, used for the treatment of patients with ALL, we examined the effect of this extract in combination with Prednisolone after 24 and 48 h incubation, using the MTT assay. To this end, the NALM-6 and REH cells were treated with the desired concentration of the extract and Prednisolone alone and in combination with each other, and their metabolic activity was investigated after the incubation period. The use of Prednisolone alone reduced the survival of NALM-6 cells in dose- and time- dependent manner, whereas, it had no cytotoxic effect on REH cells as a glucocorticoid-resistant cells (Figure 6). For synergic analysis, different concentration of *B. aspera* extract in combination with 500 nM and 1 M Prednisolone, which were lower than the maximum concentration of Prednisolone in plasma^   4 ^, were used. To evaluate the effect of the *B. aspera* extract in combination with Prednisolone, the combination index (CI) method was used. It is one of the most common methods for evaluating drug interactions in combination chemotherapy, which CI<1, CI>1, and CI=1 indicate synergistic, antagonistic, and additive effects between two drugs^   16 ^. The values of CI obtained from the effect of *B. aspera* extract in combination with Prednisolone were calculated with CompuSyn software. The obtained CI was almost equal to 1, suggesting that *B. aspera* extract did not induce greater cytotoxic effect as compared to using either drug alone and had additive effect.

## Discussion

 Plants have provided a prime source of highly effective drugs for the treatment of many diseases^17^. Nowadays, many clinically approved or under trial anticancer medications are derived from nature^   18 ^. In this study, we observed that the extract of aerial parts of *B. aspera* plant induces a significant dose- and time-dependent cytotoxic effect on the acute lymphoblastic leukemia cell lines, namely NALM-6 and REH. Interestingly, we found lesser cytotoxic effect by different doses of this extract on non-cancerous cell line (MDBK), which is consistent with a study by Sahranavard et al. who reported that the extract from the root of *B. aspera* has a cytotoxic effect on the breast cancer cell line (MCF-7) and they found a weaker cytotoxic effect on MDBK cells^   9 ^. Additionally, in this study, the synergistic effects of *B. aspera* extract were investigated in combination with Prednisolone, the most used glucocorticoid^   19 ^, and no synergistic effect was seen in BCP-ALL cell lines (CI1).

In the next stage, the apoptotic effects of *B. aspera* extract on two BCP-ALL cell lines were evaluated. Apoptosis plays an axial role in cancer and its induction in cancer cells is essential to achieve a successful treatment^   20 ^. Our results showed that the *B. aspera* extract causes cell death in BCP-ALL cell lines (NALM-6 and REH) through apoptosis induction, which increases in dose-dependent manner. In line with our findings, Pourgonabadi et al. reported that the *B. aspera* root extract triggers apoptosis in HN-5 (head and neck squamous cell carcinoma) and Hela (cervix adenocarcinoma) cell lines^   11 ^. Apoptosis is highly regulated and conserved cellular process^   21 ^ controlled by regulators, which have either an inhibitory effect on programmed cell death (anti-apoptotic) or block the protective effect of inhibitors (pro-apoptotic)^   22 ^. Among the intracellular factors, the balance between *Bax* (powerful activator of apoptosis) and *Bcl-2* (anti-apoptotic counterpart of *Bax*) is known as the most significant parameter in cell survival^15^. Therefore, the mRNA expression levels of *Bax* and *Bcl-2* were investigated to evaluate the effect of the *B. aspera* extract on the induction of cell death. Our gene expression study demonstrated that the extract of *B. aspera* increases the expression of *Bax* and reduces the expression of *Bcl-2* in BCP-ALL cell lines. These findings indicate the disruption of balance between pro- and anti-apoptotic proteins in favor of pro-apoptotic proteins, which finally leads to the induction of apoptosis. Moreover, the relative activity of caspase-3, as the main caspase in common pathway in apoptosis, was increased in cells treated with *B. aspera* extract. In Benarba et al. study, it was reported that the extract of *B. dioica* (a species of Bryonia genus) results in the induction of apoptosis in Burkitt’s lymphoma cell line BL41 through the intrinsic pathway and increasing the activity of caspase-3^   23 ^. In another study, it was shown that the extract of *B. laciniosa* (a species of Bryonia genus) triggers the induction of apoptosis through increasing the activity of caspase-3 and caspase-8 in MCF-7 (human breast adenocarcinoma) and SiHa (human squamous cell carcinoma; cervix) cell lines^24^. In general, it can be concluded that the extract of *B. aspera* causes the inhibition of *Bcl-2* and dimerization of *Bax* that trigger the release of cytochrome c from the mitochondria and activation of caspase-3, and this phenomenon finally induces cell death. 

In summary, our study demonstrates that the *B. aspera* extract can induce apoptosis in BCP-ALL cell lines. However, further investigations, including clinical trials and a detailed understanding of the *B. aspera* extract underlying mechanism of action are warranted to determine the efficacy of this natural agent.

## CONCLUSION

 By and large, our present findings provide evidence that the *B. aspera* extract has anti-leukemic properties, and the mechanism of action is likely to be dependent on transcriptional regulation of apoptotic signaling proteins. Thus, *B. aspera* could be considered as promising source of developing novel therapeutics against acute lymphoblastic leukemia.
